# Severity of oxidative stress as a hallmark in COVID-19 patients

**DOI:** 10.1186/s40001-023-01401-2

**Published:** 2023-12-04

**Authors:** Alireza Bastin, Fatemeh Abbasi, Narges Roustaei, Jahangir Abdesheikhi, Hossein Karami, Mohammad Gholamnezhad, Mahdieh Eftekhari, Amirhossein Doustimotlagh

**Affiliations:** 1 Clinical Research Development Center, “The Persian Gulf Martyrs” Hospital, Bushehr University of Medical Sciences, Bushehr, Iran; 2 Department of Clinical Biochemistry, Faculty of Medicine, Bushehr University of Medical Sciences, Bushehr, Iran; 3 Department of Infectious Disease, Faculty of Medicine, Bushehr University of Medical Sciences, Bushehr, Iran; 4 Department of Biostatistics and Epidemiology, School of Health, Yasuj University of Medical Sciences, Yasuj, Iran; 5 Department of Clinical Immunology, School of Medicine, Bushehr University of Medical Sciences, Bushehr, Iran; 6 Department of Infectious Disease, Faculty of Medicine, Yasuj University of Medical Sciences, Yasuj, Iran; 7 Pharmaceutical Sciences Research Center, Kermanshah University of Medical Sciences, Kermanshah, Iran; 8 Department of Pharmacognosy and Pharmaceutical Biotechnology, Faculty of Pharmacy, Kermanshah University of Medical Sciences, Kermanshah, Iran; 9 Medicinal Plants Research Center, Yasuj University of Medical Sciences, Yasuj, Iran; 10 Department of Clinical Biochemistry, Faculty of Medicine, Yasuj University of Medical Sciences, Yasuj, Iran

**Keywords:** COVID-19, Oxidative stress, Antioxidant, MDA, SOD, G6PD

## Abstract

**Introduction:**

Understanding the mechanisms and identifying effective treatments for the COVID-19 outbreak are imperative. Therefore, this study aimed to assess the antioxidant status and oxidative stress parameters as potential pivotal mechanisms in asymptomatic, non-severe, and severe COVID-19 patients.

**Methods:**

This study is a case–control study that was performed on patients referred to the Persian Gulf Martyrs Hospital of Bushehr University of Medical Sciences, Bushehr, Iran, from May 2021 to September 2021. A total of 600 COVID-19 patients (non-severe and severe group) and 150 healthy volunteers of the same age and sex were selected during the same period. On the first day of hospitalization, 10 ml of venous blood was taken from subjects. Then, hematological, biochemical, serological, antioxidant and oxidative stress parameters were determined.

**Results:**

Our results indicated that ESR, CRP, AST, ALT, and LDH significantly augmented in the severe group as compared to the non-severe and normal groups (*P* ≤ 0.05). It was observed that the levels of FRAP, G6PD activity, and SOD activity significantly reduced in the non-severe patients in comparison with the severe and normal groups (*P* ≤ 0.05). We found that MDA content and NO metabolite markedly increased in severe patients as compared to the non-severe group.

**Conclusions:**

Taken together, it seems that the balance between antioxidants and oxidants was disturbed in COVID-19 patients in favor of oxidant markers. In addition, this situation caused more aggravation in severe patients as compared to the non-severe group.

**Supplementary Information:**

The online version contains supplementary material available at 10.1186/s40001-023-01401-2.

## Introduction

The coronavirus, as one of the most important pathogens, targets the respiratory system [1]. Prevalence of previous coronaviruses such as SARS (SARS) and Middle East respiratory syndrome (MERS) have been identified as an important health threats in recent years [2]. At the end of 2019, a new virus from this family was identified in Wuhan, China [3]. COVID-19 can damage organs, such as the lung, heart, liver, kidneys, immune system, and blood. It is necessary to pay attention to possible multi-organ damage and its protection and prevention [4].

COVID-19 can not only cause pneumonia but can also damage other organs, such as the heart, liver, kidneys, immune system, and blood. Patients eventually die from multiple organ failure, shock, acute respiratory distress syndrome, heart failure, arrhythmia, and renal failure. Therefore, it is necessary to pay attention to possible multi-organ damage and its protection and prevention in the treatment of this disease [5].

Oxidative stress is found in many chronic diseases, such as diabetes, cancer, coronary heart disease, etc., and some infections [6]. Excessive production of reactive oxygen and nitrogen species (ROS and RNS) through oxidation and nitrification of various biological targets within the cell causes inflammation and exacerbates the disease process, resulting in damage to various organs [7].

In a healthy cell, there is a good balance between pro oxidants and antioxidants. With an increase in pro-oxidants or a decrease in antioxidants, oxidative stress occurs, which, if prolonged, can cause serious cell damage. Excessive formation of reactive oxygen species (ROS) can induce oxidative stress and lead to cell damage that can lead to cell death [8]. The body contains a complex antioxidant defense network that relies on endogenous and non-enzymatic enzymatic antioxidants. Important non-enzymatic sources of antioxidants include vitamins A, C, and E, and compounds, such as beta-carotene, and metabolites, such as glutathione [9]. Antioxidant enzymes also play an important role; the most important of these enzymes are catalase (CAT), glutathione reductase (GR), glutathione peroxidase (GPx), and superoxide dismutase (SOD) [10]. As mentioned, an imbalance between free radicals (oxidants) and antioxidant systems causes oxidative stress. The cell can tolerate mild oxidative stress, but in more severe cases, the cell membrane is damaged, and subsequent pathological complications such as lipid peroxidation occur [11]. Reactive oxygen species (ROS) can interact with cellular components, such as lipids, proteins, and DNA through certain reactions [12]. Among these molecules, lipid peroxidation is more harmful, because the formation of lipid peroxidation products directly promotes free radical reactions. Excessive oxidation of lipids alters the physical properties of cell membranes and can cause covalent changes in proteins and nucleic acids [13, 14]. The most common of these changes are the oxidation of thiol proteins and the formation of carbonyl proteins, which occur mainly on the amino acids cysteine and methionine. Oxidative changes in proteins can inhibit the binding of the substrate, the quality of the activity of enzymes, and also reduce their activity [15].

Some articles have suggested that oxidative stress may play an important role in activating acute inflammation during SARS-CoV-2 infection. Respiratory viral infections are generally associated with cytokine production, inflammation, cell death, and other pathophysiological processes that may be associated with redox imbalance or oxidative stress [16]. On the other hand, antioxidant molecules such as glutathione (GSH) [17], alpha lipoic acid (ALA) [18], *N*-acetylcysteine (NAC) [19], vitamin D [20], vitamin C [21], vitamin E [22], and some members of the vitamin B family [23, 24] play a key role in maintaining antioxidant balance and reducing oxidative damage caused by SARS-CoV-2 [6].

Current research on nucleated cells shows that G6PD is involved in an assortment of cellular processes through redox signaling. A close relationship has been shown between G6PD-derived NADPH and reactive species [25]. Our previous study suggested that the consumption of antioxidants such as polydatin may be effective in reducing oxidative stress and inflammation in patients with COVID-19 [26].

Since the outbreak of COVID-19, which has become a global epidemic, there is an urgent need to understand the mechanisms of this disease and thus identify effective treatments. Therefore, the purpose of the current study was to evaluate the antioxidant status (including GSH content and total plasma antioxidant capacity) and oxidative stress parameters (including malondialdehyde and nitric oxide) as possible key mechanisms in asymptomatic, non-severe, and severe COVID-19 patients.

## Materials and methods

### Patient selection

This study is a case–control study that was performed on patients referred to the Persian Gulf Martyrs Hospital of Bushehr University of Medical Sciences, Bushehr, Iran, from May 2021 to September 2021. A total of 600 COVID-19 patients who had been confirmed by a specialist physician using a real-time PCR test on nasopharyngeal samples or CT scans were included in the study. In addition, 150 healthy volunteers of the same age and sex were selected during the same period. Healthy subjects were confirmed to have no underlying disease. Individuals with a history of diabetes, hypertension, cancer, severe liver disease, renal insufficiency (GFR < 60 mL/min), any form of renal replacement therapy, pregnancy, or autoimmune disorders were excluded from both the control and patient groups. In addition, subjects were excluded from the study if they had a special diet or took antioxidant supplements, such as vitamin C, vitamin E, selenium, etc.

COVID-19 patients were divided into two groups based on clinical manifestations: the first group of patients had mild to moderate COVID-19 infection and symptoms, such as clinical or radiographic evidence of lower respiratory tract disease and oxygen saturation ≥ 94 (*n* = 300), and the second group of patients with severe COVID-19 infection and symptoms such as oxygen saturation < 94%, respiratory rate ≥ 30 breaths/min, and lung infiltration > 50% (*n* = 300) were selected. The protocol of this study was approved by Bushehr University of Medical Sciences with the code 9906113797 and the ethical code: IR.BPUMS.REC.1399.189. Conscious consent was obtained from all subjects before entering the study and performing the experiments.

### Samples and data collection

Demographic information was recorded through interviews with participants. On the first day of hospitalization, 10 ml of venous blood was taken from subjects and added to tubes with EDTA anticoagulant and sodium citrate for routine hematology tests and tubes without anticoagulation to measure serological, biochemical, and oxidative stress parameters. Blood samples were centrifuged at 2500 rpm for 10 min, and serums were stored at −20 °C after separation to measure serological, biochemical, and oxidative stress analyses. After collecting the samples, CBC parameters were performed with an automatic hematological autoanalyzer and ESR was performed automatically with an ESR analyzer. Evaluation of biochemical and serological parameters was performed using standard kits (Pars Azmoun, Tehran, Iran) and a biochemical automatic analyzer Dirui CS-400 (Dirui, Changchun, China). The level of G6PD enzyme was determined using the Pars Azmoon kit and using the Biotecnica BT-3500 chemistry analyzer (Diamond Diagnostics, Holliston, MA, USA).

### Measurement of oxidative stress markers

#### Measurement of malondialdehyde (MDA)

One of the indicators of lipid peroxidation is the evaluation of malondialdehyde (MDA) levels. According to our previous study [27], MDA levels were measured based on the TBA reaction. Briefly, 100 μl of patient serum was mixed in a reagent containing 15% w/v TCA, 0.375% w/v TBA, and 0.25 N HCl and then measured at 535 nm. Finally, the level of MDA was expressed in µmol/L.

### Nitric oxide metabolite

Nitrite level was measured as a nitric oxide indicator by the Griess method [28]. First, patients' serum was deproteinized with acetonitrile, and then 100 μl of supernatant was added to the Griess reagent. After incubation for 30 min, the absorbance of the samples was read at 540 nm. Sodium nitrite was also used as a standard, and the level of nitric oxide metabolite was calculated using a standard curve.

### Total antioxidant capacity

Total serum antioxidant capacity was measured by the ferric reducing antioxidant power (FRAP) method [29]. Total antioxidant capacity is expressed by the method of determining the antioxidant/regenerative power of ferric. At low pH, the ferric–TPTZ complex is reduced to ferrous form, and its concentration in blue was measured at 593 nm. The standard curve was drawn based on standard solutions of ferrous sulfate. The change in absorption is directly related to the total antioxidant-reducing power of the complex reaction. FRAP content is expressed in µmol/L.

### Measuring the antioxidant enzyme activity

Measurement of superoxide dismutase activity was performed using a commercial Ransod kit (Randox, Crumlin, UK). Serum SOD activity was measured according to the previous study [30] spectrophotometry. In this protocol, SOD prevents the reduction of iodonitrotrazole (INT) in the presence of xanthine oxidase and xanthine. Then, at a wavelength of 505 nm, they were read, and their activity was expressed in U/mg.

### Statistical analysis

Entry and analysis of all data were done using a statistical software package (SPSS for Windows, Version 16.0, SPSS Inc., Chicago, IL). At first, the Shapiro–Wilk statistical test was used to assess the normality assumption. Data were presented as mean ± SD when distributed normally and as median with interquartile range (IQR) if the distribution was skewed. For comparisons among groups, if the data had a normal distribution, they were analyzed by ANOVA (Tukey’s post-hoc test) and Pearson correlation, and Kruskal Wallis (Bonferroni correction for pairwise comparisons) and Spearmen correlation if the data did not have a normal distribution. The Chi-square test compared qualitative variables among three study groups. In all tests, the significance level was set at 0.05. A simple binomial logistic regression analysis was used to assess the effect of variables on the severity of diseases. A multiple binomial logistic regression analysis was then performed, including all variables that had a *p* value in the simple logistic regression analysis < 0.2. The multiple regression analyses, *p* value, odds ratio (OR), and 95% confidence intervals (CI) for each variable were reported.

## Results

### Socio-demographic characteristics

**Fig. 1:**
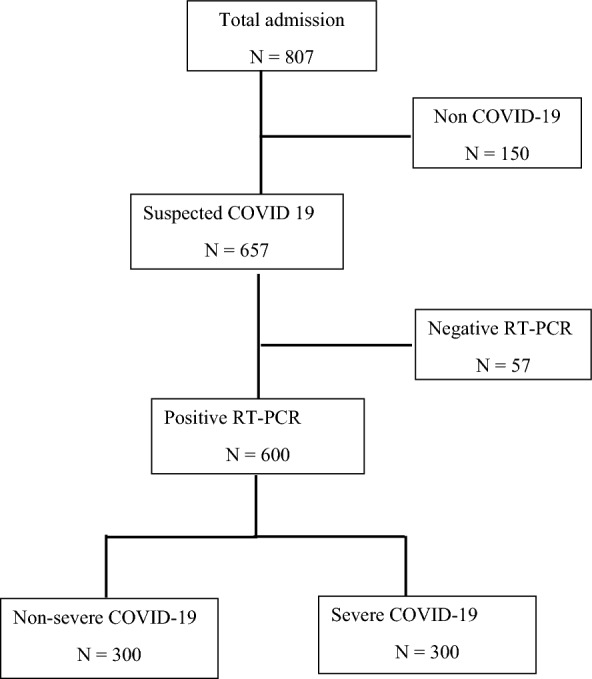
Schematic flow diagram of the study

The flow diagram of this study is shown in Fig. 1. The mean [± standard deviation (SD)] age of COVID-19 patients, including normal, non-severe, and severe groups, in this study were 37.29 ± 10.63, 39.55 ± 9.84, and 40.37 ± 9.99 years, respectively. There was a significant change between normal and severe group (*P* ≤ 0.05). Male-to-female percent of normal, non-severe, and severe group in the current study were 51.7/48.3%, 56.0/44.0%, and 43.1/56.9%, respectively (Table 1).

**Table 1:** Patient demographic information and clinical characteristics

	Normal	Non-severe	Severe	*p* value
Age (years)	37.29 ± 10.63	39.55 ± 9.84	40.37 ± 9.99	0.009
Gender				
Male	78 (51.7%)	168 (56%)	131 (43.1%)	0.025
Female	73 (48.3%)	132 (44%)	173 (56.9%)	

### Hematological and biochemical parameters in different stages of COVID-19 patients

**Fig. 2:**
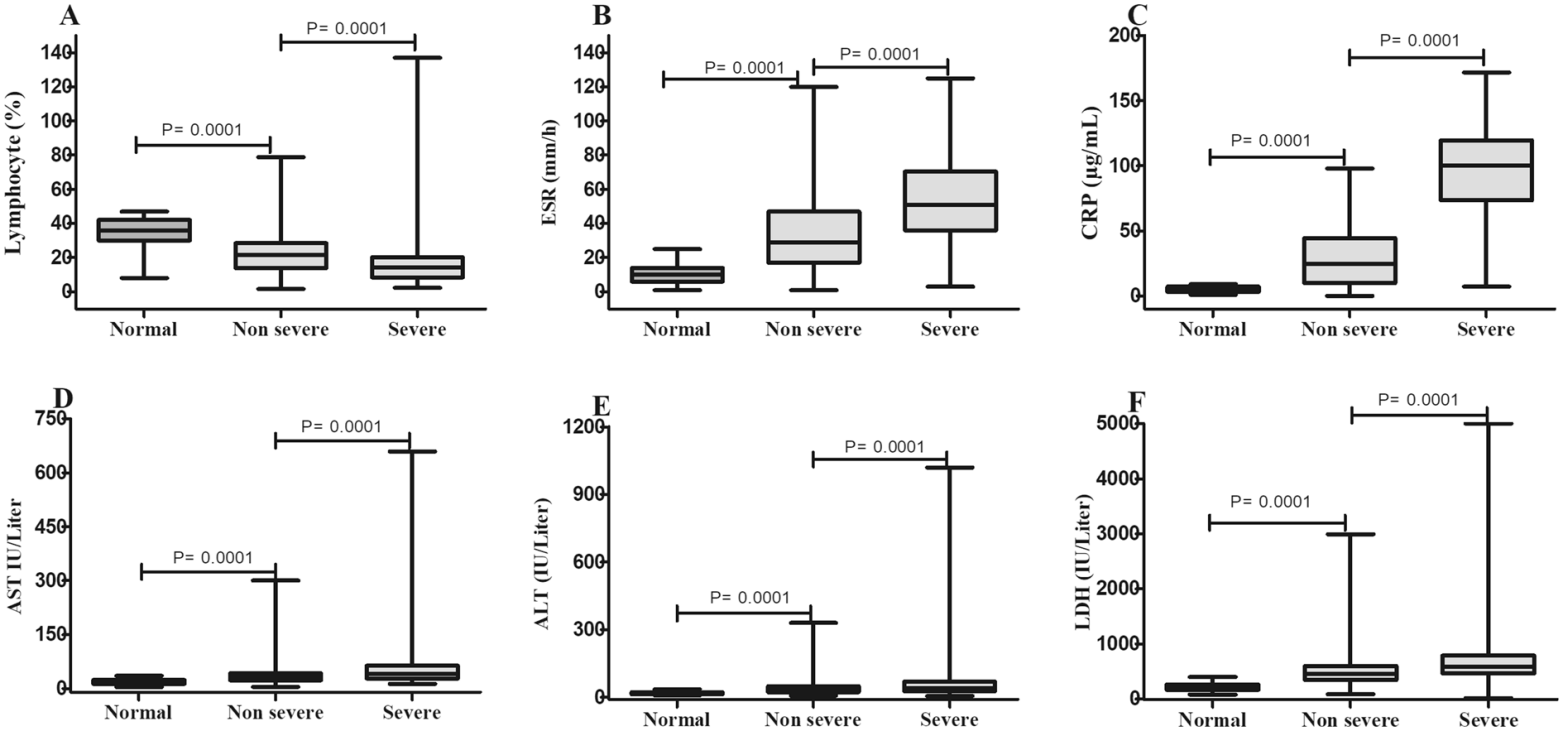
Levels of biochemical and hematological parameters between groups. **A** Lymphocyte count; **B** ESR; **C** CRP; **D** AST; **E** ALT; and **F** LDH. *p < 0.01, in comparison with normal group. #*p* < 0.01, in comparison with non-severe group

**Fig. 3:**
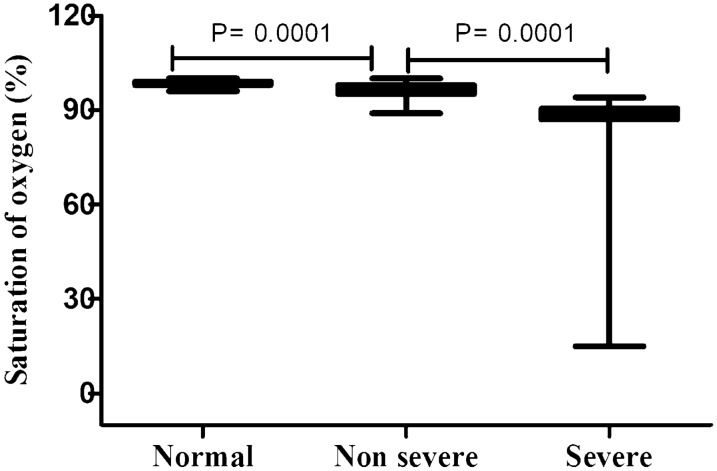
Levels of saturation of oxigen between groups. *p < 0.05, in comparison with normal group. #*p* < 0.05, in comparison with non-severe group

Our results indicated that ESR, CRP, AST, ALT, and LDH significantly increased in the non-severe group in contrast to the normal group (*P* ≤ 0.01). Moreover, these markers were significantly augmented in the severe group as compared to non-severe patients (*P* ≤ 0.01). In addition, the lymphocyte count markedly declined gradually in COVID-19 patients with different disease severity (*P* ≤ 0.01); the severe group had the lowest level of it (Fig. 2). The saturation of oxygen was markedly reduced in the normal and non-severe groups as compared to the severe group (*P* ≤ 0.01) (Fig. 3). Additional file 1: Table S1 shows other parameters between groups (Table 2).

**Table 2:** Multiple logistic regression to assess the effect of variables on the severity of diseases

Variables	B	S.E	Wald	df	*p* value	OR	95% CI for OR	
							Lower	Upper
Age	−0.001	0.019	0.005	1	0.942	0.999	0.962	1.036
M/F(1)	0.600	0.450	1.777	1	0.182	1.823	0.754	4.405
WBC	−0.033	0.064	0.269	1	0.604	0.967	0.854	1.096
RBC	0.152	0.362	0.177	1	0.674	1.165	0.573	2.367
Hb	−0.173	0.131	1.731	1	0.188	0.841	0.651	1.088
LYM	0.024	0.050	0.226	1	0.634	1.024	0.928	1.130
Net	0.043	0.042	1.025	1	0.311	1.044	0.961	1.134
ESR	0.018	0.011	2.756	1	0.097	1.018	0.997	1.039
CRP	0.067	0.009	54.011	1	< 0.001	1.069	1.050	1.088
PTT	0.002	0.010	0.037	1	0.848	1.002	0.982	1.022
BS	0.015	0.004	12.238	1	< 0.001	1.016	1.007	1.024
BUN	0.000	0.004	0.003	1	0.955	1.000	0.993	1.008
Cra	−0.093	0.237	0.155	1	0.694	0.911	0.572	1.451
Bili.d	−1.242	1.161	1.143	1	0.285	0.289	0.030	2.814
Bili.T	0.767	1.037	0.547	1	0.459	2.153	0.282	16.434
AST	0.003	0.011	0.077	1	0.782	1.003	0.982	1.024
ALT	−0.002	0.007	0.058	1	0.810	0.998	0.986	1.011
CPK	0.001	0.001	1.212	1	0.271	1.001	1.000	1.002
LDH	0.000	0.001	0.005	1	0.942	1.000	0.998	1.002
Alb	−0.452	0.463	0.952	1	0.329	0.636	0.257	1.577
K	0.075	0.428	0.031	1	0.861	1.078	0.466	2.494
G6PD	−0.009	0.048	0.036	1	0.850	0.991	0.901	1.089
FRAP	−0.002	0.001	4.136	1	0.042	0.998	0.996	1.000
NO	0.078	0.053	2.194	1	0.139	1.081	0.975	1.199
MDA	0.704	0.105	45.032	1	< 0.001	2.022	1.646	2.483
SOD	−0.063	0.028	4.836	1	0.028	0.939	0.888	0.993
Constant	−11.713	5.458	4.605	1	0.032	0.000		

### A disturbed oxidant–antioxidant balance is associated with increased disease severity

**Fig. 4:**
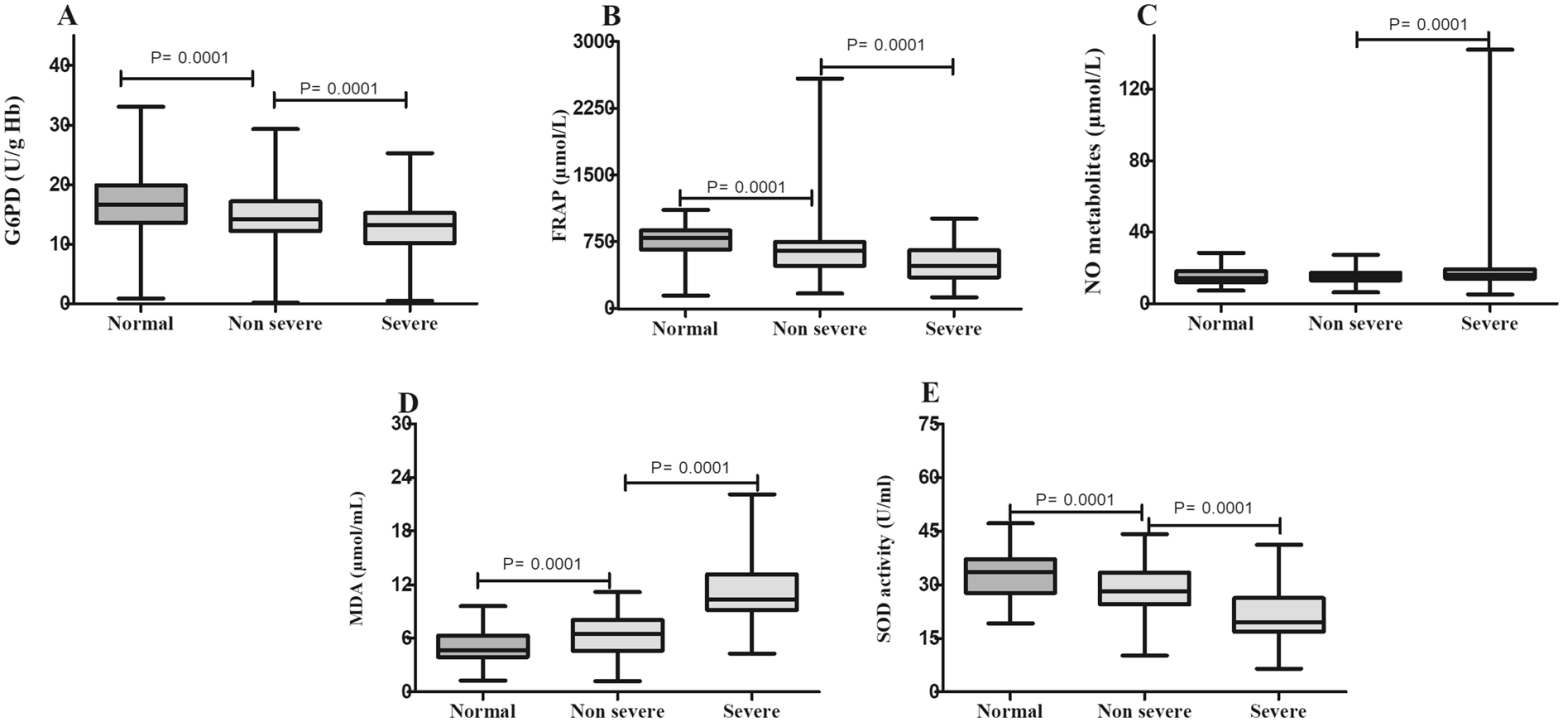
Levels of oxidative stress parameters between groups **A** G6PD; **B** FRAP; **C** NO metabolite; **D** MDA; and **E** SOD. *p < 0.0001, in comparison with normal group. #*p* < 0.0001, in comparison with non-severe group

Figure 4 shows comparisons of G6PD, FRAP, NO metabolite, MDA, and SOD levels between groups. It was observed that the levels of FRAP, G6PD activity, and SOD activity were significantly reduced in non-severe patients in comparison with the normal group (*P* ≤ 0.0001). Furthermore, these parameters markedly decreased in the non-severe group as compared to severe patients (*P* ≤ 0.0001). We found that patients with different degrees of COVID-19 showed a gradual increase in MDA content, with the lowest level found in the normal group and the highest level observed in the severe group. It showed that the levels of NO metabolite significantly increased in severe patients in comparison with the normal and non-severe groups (*P* ≤ 0.0001). However, NO metabolite level showed no significant change between the normal and non-severe groups.

### Analysis of correlations between studied parameters

We found that there is a statistically significant positive correlation between the saturation of oxygen and LYM count (*r* = 0.46, *P* < 0.0001), the saturation of oxygen and SOD activity (*r* = 0.52, *P* < 0.0001), as well as plasma MDA and CRP levels (*r* = 0.66, *P* < 0.0001). However, negative correlations were observed between the saturation of oxygen and CRP (*r* = −0.80, *P* < 0.0001), ESR (*r* = −0.50, *P* < 0.0001), and MDA (*r* = −0.60, *P* < 0.0001) content, as well as plasma MDA and SOD activity (*r* = −0.40, *P* < 0.0001) (Additional file 1: Table S1).

The negative correlations were observed between the death and LYM count (*r* = −0.27, *P* < 0.0001), SOD activity (*r* = −0.24, *P* < 0.0001), as well as the saturation of oxygen (*r* = −0.32, *P* < 0.0001). However, a positive correlation was observed between the death and CRP (*r* = 0.27, *P* < 0.0001), AST (*r* = 0.26, *P* < 0.0001), LDH (*r* = 0.32, *P* < 0.0001), and MDA level (*r* = 0.32, *P* < 0.0001) (Additional file 1: Table S1).

### Analysis of multiple logistic regression

For one unit increase in CRP, the chance of patients being in the severe group versus the non-severe group increased by approximately 6%, and this value was statistically significant (OR = 1.069; *P* < 0.001). In addition, for one unit increase in BS, the chance of patients being in the severe group significantly increased by approximately 2% (OR = 1.016; *P* < 0.001). The chance of being in the severe group was twice that of the non-severe group per one-unit increase in MDA (OR = 2.022; *P* < 0.001). In addition, for one unit decrease in FRAP, the chance of patients being in the severe group significantly decreased by 0.2% (OR = 0.998; *P* = 0.042). For one unit decrease in SOD, the chance of patients being in the severe group significantly decreased by approximately 0.6% (OR = 0.939; *P* = 0.028).

## Discussion

Various studies have shown that COVID-19 patients is involved in inducing oxidative stress and inhibiting the activity of the body's antioxidant system [31, 32]. Our findings in this study revealed that COVID-19 patients have low levels of antioxidant parameters such as TAC, SOD and high serum levels of oxidative stress markers, such as MDA.

The imbalance between oxidant production and antioxidant mechanisms leads to oxidative stress, which can lead to oxidative damage, such as lipid peroxidation and DNA oxidation [33]. Malondialdehyde, which is derived from lipid peroxidation, is one of the best indicators of oxidative stress [34, 35]. We only found a statistically significant positive correlation between plasma MDA and CRP levels. It seems that MDA and CRP levels in COVID-19 patients increased significantly, which is proportional to the severity of the disease. Viruses induce oxidative stress to facilitate their proliferation within the cell [36]. Reactive oxygen species (ROS) are produced by pulmonary alveolar macrophages in response to COVID-19 infection [37]. Extensive production of ROS and failure to neutralize it by the body's defense mechanisms, including innate immunity and antioxidant defense, following viral infection leads to a wide range of pathologies [38]. In some cases, viruses have the ability to suppress the Nrf2 (a transcription factor that regulates the expression of genes responsible for the production of antioxidant proteins) pathway [39, 40]. The expression of Nrf2 target genes, including homoxygenase-1 (HO-1), SOD1, SOD3, glutathione S-transferase (GST), catalase (CAT), and glutathione peroxidase (GPX) decreases [39]. Therefore, these findings confirm that oxidative stress plays an effective role in the response to the SARS-CoV-2 viral infection. In line with our finding, previous studies [41, 42] depicted that SARS-CoV-2 infection increases oxidative stress, and oxidative stress also plays a role in exacerbating the infection.

Viral infections are associated with decreased antioxidant defenses [43]. According to our results, SOD activity and TAC were significantly reduced in these patients. SOD is one of the main enzymes in the antioxidant defense system. Decreased SOD expression can increase the production of ROS and thus increase pro-inflammatory responses [44]. In general, an excessive inflammatory response is also associated with the severity of SARS-CoV-2 infection [44]. In line with our results, Lin et al. reported that H5N1 influenza infection in lung epithelial cells reduced SOD expression at mRNA and protein levels [45].

Nitric oxide is a gaseous free radical that is involved in regulating immune responses [46]. According to our data, a significant decrease in NO was observed in patients. Vasodilation effected by NO may potentially diminish lung injuries due to COVID-19 [46]. NO inhalation or a nitrate-rich diet can be useful in reversing pulmonary hypertension and mortality caused by COVID-19 [47, 48]. Decreased or impaired NO metabolism is associated with the intensity of COVID-19 disease [47]. Decreasing NO and increasing oxidative stress disrupt endothelial function and macrophages, and increases inflammatory cytokines such as tumor necrosis factor (TNF) and monocyte–absorption protein (MCP-1) in endothelial cells and monocytes [49, 50].

NO production is positively associated with G6PD activity. G6PD deficiency reduces the amount of NADPH required to form NO [37].

G6PD deficiency has a positive association with the severity of COVID-19 disease [51]. G6PD may play a substantial role in viral infections. G6PD deficiency enhances cytopathic effects and viral replication [52, 53]. According to the results of our study, the level of G6PD in these patients was significantly reduced. NADPH, the main product of G6PD, is vital for the regeneration of GSH, which plays an important role in cellular antioxidant defense [54]. NADPH also plays a variety of roles in cellular regulation by redox signals, for example, ROS and RNS, which produce NADPH oxidase (NOX) and nitric oxide synthase (NOS), respectively [54]. G6PD maintains redox homeostasis by keeping cytotoxic ROS at appropriate levels, because high levels of ROS are cytotoxic [55]. G6PD is involved in modulating the inflammatory response in immune cells. Peripheral mononuclear cells produce lower levels of proinflammatory cytokines, IL-6 and IL-1β, under G6PD deficiency [56]. G6PD deficiency affects many cellular immune responses, such as increased production of the pro-inflammatory cytokine IL-8 and impaired inflammasome activation [57, 58]. In addition, G6PD deficiency has been shown to increase viral infections. During the current COVID-19 epidemic, G6PD deficiency has exacerbated the severity of the infection [59].

In laboratory findings in COVID-19 patients, the number of lymphocytes and percentage of oxygen saturation decreased, but the levels of liver enzymes (AST and ALT), inflammatory markers (CRP and ESR), and lactate dehydrogenase increased. Important causes of lymphocyte deficiency in COVID-19 patients include: (a) because lymphocytes express the coronavirus receptor called ACE2, SARS-CoV-2 can directly infect lymphocytes and lead to lymphocyte death [60]. (B) Increased inflammatory cytokines induced by SARS-CoV-2 may lead to increased lymphocyte apoptosis compared to other viruses [61]. Thus, a decrease in lymphocytes may eventually reduce the host's antiviral immunity, which causes infection.

Coagulation, sepsis, and decreased oxygen transport to tissues are significant symptoms in COVID-19 patients [62]. According to our results, the percentage of oxygen saturation in these patients showed a significant decrease. Hypoxia in tissues causes the production of ROSs, such as superoxide and H_2_O_2_, which increase the expression of inflammatory cytokines [63, 64]. These inflammatory cytokines aggravate the infection in COVID-19 patients by increasing oxidative stress.

Serum aminotransferase levels are effective indicators of hepatocellular injury [65]. The results of our study showed an increase in hepatic aminotransferase such as AST and ALT in COVID-19 patients compared to the control group. Hepatocytes and bile duct cells over express the ACE2 receptor, which binds to the SARS-CoV-2 virus [66]. This ACE2 receptor could be one of the receptors involved in the liver damage seen in COVID-19 [67]. Similar to our results, a study by Medetalibeyoglu et al. showed that an increase in hepatic aminotransferases such as AST and ALT was associated with a more severe course and an increase in mortality in COVID-19 patients [68].

Some cytokines, such as hepatocyte growth factor (HGF), play an important role in the severity of COVID-19 [69]. Increased inflammatory mediators play an important role in pneumonia caused by human pathogenic coronaviruses, including SARS-CoV-2 [44]. In this study, high levels of the two inflammatory markers CRP and ESR were associated with the severity of COVID-19, thus confirming the results of previous studies [70, 71]. Recent research demonstrated that patients with CRP > 64.75 mg/L were more likely to have severe complications [72, 73]. Alamdari et al. [7] showed an increase in the oxidative stress level of COVID-19 patients as well as a significant relationship between CRP and nitrite levels in the inflammatory phase. Moorthy et al. [74], depicted that the CRP, LDH, eosinophil, and lymphocyte counts serve to predict the severity and prognosis of COVID-19 patients [74]. Some studies have shown that LDH has a poor prognosis with COVID-19 [67, 75]. According to the findings of this study, the serum level of LDH in COVID-19 patients related to the severity of the disease had a significant decrease compared to the control group. LDH has been found to affect the prognosis of different diseases, including cancer [76]. Elevated LDH in patients with COVID-19 can indicate lung and tissue damage [60]. COVID-19 may lead to inadequate tissue perfusion and multiple organ failure by various mechanisms, including thrombosis, which increases LDH [77]. Thus, high LDH serves as a biomarker of the disease expansion.

Our study has some limitations. First, patients with mild symptoms may not visit the clinics and only stay at home. This may potentially increase the ratio of patients with drastic illness in our study; second, most of our patients were admitted to the hospital only when they developed symptoms of COVID-19. This diagnostic criterion may underestimate the actual population of COVID-19 patients.

### Supplementary Information

**Additional file 1: Table S1.** Levels of biochemical and hematological parameters between groups. **Table S2.** Statistical significant correlations between studied parameters.

## Data Availability

Data are contained within the article.

## References

[1] Zhang Y, Geng X, Tan Y, Li Q, Xu C, Xu J (2020). New understanding of the damage of SARS-CoV-2 infection outside the respiratory system. Biomed Pharmacother.

[2] Liu S, Chan T-C, Chu Y-T, Wu JT-S, Geng X, Zhao N (2016). Comparative epidemiology of human infections with Middle East respiratory syndrome and severe acute respiratory syndrome coronaviruses among healthcare personnel. PLoS ONE.

[3] She J, Jiang J, Ye L, Hu L, Bai C, Song Y (2020). 2019 novel coronavirus of pneumonia in Wuhan, China: emerging attack and management strategies. Clin Transl Med.

[4] Eftekhari M, Enayati A, Doustimotlagh AH, Farzaei MH, Yosifova AI (2021). Natural products in combination therapy for COVID-19: QT prolongation and urgent guidance. Nat Prod Commun.

[5] Wang T, Du Z, Zhu F, Cao Z, An Y, Gao Y (2020). Comorbidities and multi-organ injuries in the treatment of COVID-19. Lancet.

[6] Suhail S, Zajac J, Fossum C, Lowater H, McCracken C, Severson N (2020). Role of oxidative stress on SARS-CoV (SARS) and SARS-CoV-2 (COVID-19) infection: a review. Protein J.

[7] Alamdari DH, Moghaddam AB, Amini S, Keramati MR, Zarmehri AM, Alamdari AH (2020). Application of methylene blue-vitamin C-N-acetyl cysteine for treatment of critically ill COVID-19 patients, report of a phase-I clinical trial. Eur J Pharmacol.

[8] Poljsak B, Šuput D, Milisav I (2013). Achieving the balance between ROS and antioxidants: when to use the synthetic antioxidants. Oxid Med Cell longev.

[9] Moussa Z, Judeh ZM, Ahmed SA (2019). Nonenzymatic exogenous and endogenous antioxidants. Free Radic Med Biol.

[10] Khan MA, Tania M, Zhang D-Z, Chen H-C (2010). Antioxidant enzymes and cancer. Chin J Cancer Res.

[11] Birben E, Sahiner UM, Sackesen C, Erzurum S, Kalayci O (2012). Oxidative stress and antioxidant defense. World Allergy Organ J.

[12] Bayr H (2005). Reactive oxygen species. Crit Care Med.

[13] Gaschler MM, Stockwell BR (2017). Lipid peroxidation in cell death. Biochem Biophys Res Commun.

[14] Devasagayam T, Tilak J, Boloor K, Sane KS, Ghaskadbi SS, Lele R (2004). Free radicals and antioxidants in human health: current status and future prospects. Japi.

[15] Ahmad S, Khan H, Shahab U, Rehman S, Rafi Z, Khan MY (2017). Protein oxidation: an overview of metabolism of sulphur containing amino acid, cysteine. Front Biosci (Schol Ed).

[16] Delgado-Roche L, Mesta F (2020). Oxidative stress as key player in severe acute respiratory syndrome coronavirus (SARS-CoV) infection. Arch Med Res.

[17] Silvagno F, Vernone A, Pescarmona GP (2020). The role of glutathione in protecting against the severe inflammatory response triggered by COVID-19. Antioxidants.

[18] Dragomanova S, Miteva S, Nicoletti F, Mangano K, Fagone P, Pricoco S (2021). Therapeutic potential of alpha-lipoic acid in viral infections, including COVID-19. Antioxidants.

[19] Wong KK, Lee SWH, Kua KP (2021). N-Acetylcysteine as adjuvant therapy for COVID-19–a perspective on the current state of the evidence. J Inflamm Res.

[20] Ricci A, Pagliuca A, D’Ascanio M, Innammorato M, De Vitis C, Mancini R (2021). Circulating Vitamin D levels status and clinical prognostic indices in COVID-19 patients. Respir Res.

[21] Hiedra R, Lo KB, Elbashabsheh M, Gul F, Wright RM, Albano J (2020). The use of IV vitamin C for patients with COVID-19: a case series. Expert Rev Anti Infect Ther.

[22] Tavakol S, Seifalian AM (2021). Vitamin E at a high dose as an anti-ferroptosis drug and not just a supplement for COVID-19 treatment. Biotechnol Appl Biochem.

[23] Raines NH, Ganatra S, Nissaisorakarn P, Pandit A, Morales A, Asnani A (2021). Niacinamide may be associated with improved outcomes in COVID-19-related acute kidney injury: an observational study. Kidney360.

[24] Tan CW, Ho LP, Kalimuddin S, Cherng BPZ, Teh YE, Thien SY (2020). Cohort study to evaluate effect of vitamin D, magnesium, and vitamin B12 in combination on severe outcome progression in older patients with coronavirus (COVID-19). Nutrition.

[25] Yang H-C, Wu Y-H, Yen W-C, Liu H-Y, Hwang T-L, Stern A (2019). The redox role of G6PD in cell growth, cell death, and cancer. Cells.

[26] Doustimotlagh AH, Eftekhari M (2021). Glucose-6-phosphate dehydrogenase inhibitor for treatment of severe COVID-19: Polydatin. Clin Nutr ESPEN.

[27] Sadeghi A, Bastin AR, Ghahremani H, Doustimotlagh AH (2020). The effects of rosmarinic acid on oxidative stress parameters and inflammatory cytokines in lipopolysaccharide-induced peripheral blood mononuclear cells. Mol Biol Rep.

[28] Doustimotlagh AH, Kokhdan EP, Vakilpour H, Khalvati B, Barmak MJ, Sadeghi H (2020). Protective effect of Nasturtium officinale R. Br and quercetin against cyclophosphamide-induced hepatotoxicity in rats. Mol Biol Rep.

[29] Gheitasi I, Azizi A, Omidifar N, Doustimotlagh AH (2020). Renoprotective effects of Origanum majorana methanolic L and carvacrol on ischemia/reperfusion-induced kidney injury in male rats. Evidence-Based Complement Altern Med.

[30] Arya A, Azarmehr N, Mansourian M, Doustimotlagh AH (2021). Inactivation of the superoxide dismutase by malondialdehyde in the nonalcoholic fatty liver disease: a combined molecular docking approach to clinical studies. Arch Physiol Biochem.

[31] Cecchini R, Cecchini AL (2020). SARS-CoV-2 infection pathogenesis is related to oxidative stress as a response to aggression. Med Hypotheses.

[32] Laforge M, Elbim C, Frère C, Hémadi M, Massaad C, Nuss P (2020). Tissue damage from neutrophil-induced oxidative stress in COVID-19. Nat Rev Immunol.

[33] Park HS, Kim SR, Lee YC (2009). Impact of oxidative stress on lung diseases. Respirology.

[34] Komaravelli N, Casola A (2014). Respiratory viral infections and subversion of cellular antioxidant defenses. J Pharmacogenomics Pharmacoproteomics.

[35] Tejchman K, Kotfis K, Sieńko J (2021). Biomarkers and mechanisms of oxidative stress—last 20 Years of Research with an emphasis on kidney damage and renal transplantation. Int J Mol Sci.

[36] Gjyshi O, Bottero V, Veettil MV, Dutta S, Singh VV, Chikoti L (2014). Kaposi's sarcoma-associated herpesvirus induces Nrf2 during de novo infection of endothelial cells to create a microenvironment conducive to infection. PLoS Pathog.

[37] Jain SK, Parsanathan R, Levine SN, Bocchini JA, Holick MF, Vanchiere JA (2020). The potential link between inherited G6PD deficiency, oxidative stress, and vitamin D deficiency and the racial inequities in mortality associated with COVID-19. Free Radical Biol Med.

[38] Zhang H, Liu H, Zhou L, Yuen J, Forman HJ (2017). Temporal changes in glutathione biosynthesis during the lipopolysaccharide-induced inflammatory response of THP-1 macrophages. Free Radical Biol Med.

[39] Hosakote YM, Jantzi PD, Esham DL, Spratt H, Kurosky A, Casola A (2011). Viral-mediated inhibition of antioxidant enzymes contributes to the pathogenesis of severe respiratory syncytial virus bronchiolitis. Am J Respir Crit Care Med.

[40] Komaravelli N, Ansar M, Garofalo RP, Casola A (2017). Respiratory syncytial virus induces NRF2 degradation through a promyelocytic leukemia protein-ring finger protein 4 dependent pathway. Free Radical Biol Med.

[41] Martín-Fernández M, Aller R, Heredia-Rodríguez M, Gómez-Sánchez E, Martínez-Paz P, Gonzalo-Benito H (2021). Lipid peroxidation as a hallmark of severity in COVID-19 patients. Redox Biol.

[42] Mehri F, Rahbar AH, Ghane ET, Souri B, Esfahani M (2021). Changes in oxidative markers in COVID-19 patients. Arch Med Res.

[43] Hosakote YM, Liu T, Castro SM, Garofalo RP, Casola A (2009). Respiratory syncytial virus induces oxidative stress by modulating antioxidant enzymes. Am J Respir Cell Mol Biol.

[44] Zhang W, Zhao Y, Zhang F, Wang Q, Li T, Liu Z (2020). The use of anti-inflammatory drugs in the treatment of people with severe coronavirus disease 2019 (COVID-19): the perspectives of clinical immunologists from China. Clin Immunol.

[45] Lin X, Wang R, Zou W, Sun X, Liu X, Zhao L (2016). The influenza virus H5N1 infection can induce ROS production for viral replication and host cell death in A549 cells modulated by human Cu/Zn superoxide dismutase (SOD1) overexpression. Viruses.

[46] Yamasaki H (2020). Blood nitrate and nitrite modulating nitric oxide bioavailability: potential therapeutic functions in COVID-19. Nitric Oxide.

[47] Ignarro LJ (2020). Inhaled NO and COVID-19. Br J Pharmacol.

[48] Sobko T, Marcus C, Govoni M, Kamiya S (2010). Dietary nitrate in Japanese traditional foods lowers diastolic blood pressure in healthy volunteers. Nitric Oxide.

[49] Parsanathan R, Jain SK (2018). L-Cysteine in vitro can restore cellular glutathione and inhibits the expression of cell adhesion molecules in G6PD-deficient monocytes. Amino Acids.

[50] Parsanathan R, Jain SK (2019). Glucose-6-phosphate dehydrogenase deficiency increases cell adhesion molecules and activates human monocyte-endothelial cell adhesion: protective role of l-cysteine. Arch Biochem Biophys.

[51] Elhabyan A, Elyaacoub S, Sanad E, Abukhadra A, Elhabyan A, Dinu V (2020). The role of host genetics in susceptibility to severe viral infections in humans and insights into host genetics of severe COVID-19: a systematic review. Virus Res.

[52] Chao Y-C, Huang C-S, Lee C-N, Chang S-Y, King C-C, Kao C-L (2008). Higher infection of dengue virus serotype 2 in human monocytes of patients with G6PD deficiency. PLoS ONE.

[53] Ho H-Y, Cheng M-L, Weng S-F, Chang L, Yeh T-T, Shih S-R (2008). Glucose-6-phosphate dehydrogenase deficiency enhances enterovirus 71 infection. J Gen Virol.

[54] Yang H-C, Cheng M-L, Ho H-Y, Chiu DT-Y (2011). The microbicidal and cytoregulatory roles of NADPH oxidases. Microbes Infect.

[55] Davies KJ (1999). The broad spectrum of responses to oxidants in proliferating cells: a new paradigm for oxidative stress. IUBMB Life.

[56] Sanna F, Bonatesta RR, Frongia B, Uda S, Banni S, Melis MP (2007). Production of inflammatory molecules in peripheral blood mononuclear cells from severely glucose-6-phosphate dehydrogenase-deficient subjects. J Vasc Res.

[57] Yen W-C, Wu Y-H, Wu C-C, Lin H-R, Stern A, Chen S-H (2020). Impaired inflammasome activation and bacterial clearance in G6PD deficiency due to defective NOX/p38 MAPK/AP-1 redox signaling. Redox Biol.

[58] Yang H-C, Cheng M-L, Hua Y-S, Wu Y-H, Lin H-R, Liu H-Y (2015). Glucose 6-phosphate dehydrogenase knockdown enhances IL-8 expression in HepG2 cells via oxidative stress and NF-κB signaling pathway. J Inflamm.

[59] Beauverd Y, Adam Y, Assouline B, Samii K (2020). COVID-19 infection and treatment with hydroxychloroquine cause severe haemolysis crisis in a patient with glucose-6-phosphate dehydrogenase deficiency. Eur J Haematol.

[60] Li X, Xu S, Yu M, Wang K, Tao Y, Zhou Y (2020). Risk factors for severity and mortality in adult COVID-19 inpatients in Wuhan. J Allergy Clin Immunol.

[61] Tan L, Wang Q, Zhang D, Ding J, Huang Q, Tang Y-Q (2020). Lymphopenia predicts disease severity of COVID-19: a descriptive and predictive study. Signal Transduct Target Ther.

[62] Connors JM, Levy JH (2020). COVID-19 and its implications for thrombosis and anticoagulation. Blood.

[63] Mantzarlis K, Tsolaki V, Zakynthinos E (2017). Role of oxidative stress and mitochondrial dysfunction in sepsis and potential therapies. Oxid Med Cell Longev.

[64] Nanduri J, Yuan G, Kumar GK, Semenza GL, Prabhakar NR (2008). Transcriptional responses to intermittent hypoxia. Respir Physiol Neurobiol.

[65] Yadlapati S, Lo KB, DeJoy R, Gul F, Peterson E, Bhargav R (2021). Prevailing patterns of liver enzymes in patients with COVID-19 infection and association with clinical outcomes. Ann Gastroenterol.

[66] Chai X, Hu L, Zhang Y, Han W, Lu Z, Ke A (2019). Specific ACE2 expression in cholangiocytes may cause liver damage after. bioRxiv.

[67] Guan G-W, Gao L, Wang J-W, Wen X-J, Mao T-H, Peng S-W (2020). Exploring the mechanism of liver enzyme abnormalities in patients with novel coronavirus-infected pneumonia. Zhonghua gan zang bing za zhi= Zhonghua ganzangbing zazhi.

[68] Medetalibeyoglu A, Catma Y, Senkal N, Ormeci A, Cavus B, Kose M (2020). The effect of liver test abnormalities on the prognosis of COVID-19. Ann Hepatol.

[69] Tamayo-Velasco Á, Martínez-Paz P, Peñarrubia-Ponce MJ, De la Fuente I, Pérez-González S, Fernández I (2021). HGF, IL-1α, and IL-27 are robust biomarkers in early severity stratification of COVID-19 patients. J Clin Med.

[70] Terpos E, Ntanasis-Stathopoulos I, Elalamy I, Kastritis E, Sergentanis TN, Politou M (2020). Hematological findings and complications of COVID-19. Am J Hematol.

[71] Cai Q, Huang D, Ou P, Yu H, Zhu Z, Xia Z (2020). COVID-19 in a designated infectious diseases hospital outside Hubei Province, China. Allergy.

[72] Sadeghi-Haddad-Zavareh M, Bayani M, Shokri M, Ebrahimpour S, Babazadeh A, Mehraeen R (2021). C-reactive protein as a prognostic indicator in COVID-19 patients. Interdiscip Perspect Infec Dis.

[73] Bastin A, Shiri H, Zanganeh S, Fooladi S, Momeni Moghaddam MA, Mehrabani M (2021). Iron chelator or iron supplement consumption in COVID-19? The role of iron with severity infection. Biol Trace Elem Res.

[74] Moorthy S, Koshy T, Silambanan S (2021). Role of inflammatory and liver function markers in assessing the prognosis of patients with COVID-19. World Acad Sci J.

[75] Martha JW, Wibowo A, Pranata R (2021). Prognostic value of elevated lactate dehydrogenase in patients with COVID-19: a systematic review and meta-analysis. Postgrad Med J.

[76] Erez A, Shental O, Tchebiner JZ, Laufer-Perl M, Wasserman A, Sella T (2014). Diagnostic and prognostic value of very high serum lactate dehydrogenase in admitted medical patients. Isr Med Assoc J.

[77] Huang I, Pranata R, Lim MA, Oehadian A, Alisjahbana B (2020). C-reactive protein, procalcitonin, D-dimer, and ferritin in severe coronavirus disease-2019: a meta-analysis. Ther Adv Respir Dis.

