# Supplementary material for: Severity of oxidative stress as a hallmark in COVID-19 patients
Source: Eur J Med Res. 2023 Dec 4;28:558. doi: 10.1186/s40001-023-01401-2 (PMC10696844; doi:10.1186/s40001-023-01401-2)
Supplement: Supplementary file 1 — Additional file 1: Table S1. Levels of biochemical and hematological parameters between groups. Table S2. Statistical significant correlations between studied parameters. [file 40001_2023_1401_MOESM1_ESM.docx]

Additional file

Table S1. The levels of biochemical and hematological parameters between groups

| **Groups** | **Normal** | **Non severe** | **Severe** |
| --- | --- | --- | --- |
| **WBC** | 6.80 (5.40- 7.70) | 5.60 (4.10- 8.10) | 6.80 (5.10- 9.17) |
| **RBC** | 4.90 (4.60- 5.90) | 4.72 (4.31- 5.15) | 4.72 (4.21- 5.14) |
| **HB** | 14.30 (13.60- 14.90) | 12.80 (11.70- 14.10) | 12.80 (11.40- 14.10) |
| **HCT** | 44.20 (44.10- 46.30) | 39.20 (36.20- 42.47) | 38.75 (35.32- 42.77) |
| **MCV** | 90.83 (86.33- 94.58) | 84.58 (80.15- 97.95) | 84.35 (79.54- 88.34) |
| **MCH** | 28.90 (28.04- 30.83) | 27.81 (25.98- 29.46) | 84.35 (79.54- 88.34) |
| **MCHC** | 32.51 (30.91- 33.58) | 32.79 (31.80- 33.76) | 32.83 (31.66- 33.87) |
| **PLT** | 220.00 (178.00- 298.00) | 201.00 (151.00- 255.00) | 194.00 (149.00- 250.00) |
| **LYM** | 36.00 (30.00- 42.00) | 21.80 (13.90- 28.45) | 14.20 (8.42- 20.37) |
| **MIX** | 10.00 (8.00- 12.00) | 7.80 (5.32- 10.40) | 5.90 (3.72- 8.40) |
| **NET** | 54.00 (47.00- 60.00) | 61.45 (70.00- 79.55) | 79.75 (70.35- 86.75) |
| **ESR** | 10.00 (6.00- 14.00) | 29.00 (17.00- 47.00) | 51.00 (36.00- 70.75) |
| **CRP** | 5.00 (3.00- 7.00) | 24.60 (9.92- 44.30) | 100.00 (73.62- 119.75) |
| **PT** | 12.80 (12.40- 13.50) | 11.00 (13.49- 15.00) | 14.00 (11.00- 16.00) |
| **PTT** | 32.00 (29.00- 35.00) | 26.15 (24.00- 29.00) | 27.60 (25.00- 30.76) |
| **BS** | 84.00 (76.00- 91.00) | 99.00 (87.00- 112.00) | 121.00 (98.00- 179.50) |
| **BUN** | 14.60 (12.70- 17.40) | 14.20 (10.12- 18.87) | 15.55 (11.50- 23.37) |
| **CRA** | 0.90 (0.70- 1.10) | 1.00 (0.90- 1.20) | 1.10 (0.90- 1.30) |
| **CA** | 9.40 (8.90- 9.70) | 9.30 (8.90- 9.90) | 9.10 (8.70- 9.70) |
| **PH** | 3.90 (3.60- 4.30) | 3.55 (3.00- 4.20) | 3.60 (3.00- 4.10) |
| **BILLID** | 0.21 (0.16- 0.32) | 0.23 (0.16- 0.34) | 0.25 (0.19- 0.35) |
| **BILLIT** | 0.50 (0.39- 0.70) | 0.56 (0.41- 0.77) | 0.59 (0.42- 0.81) |
| **AST** | 18.00 (13.00- 24.00) | 30.00 (23.00- 42.75) | 41.00 (28.00- 64.00) |
| **ALT** | 19.00 (14.00- 23.00) | 33.00 (23.00- 48.50) | 40.00 (26.00- 70.00) |
| **ALK** | 198.00 (173.00- 244.00) | 196.50 (159.00- 252.75) | 191.00 (176.25- 248.00) |
| **CPK** | 124.00 (96.00- 165.00) | 117.50 (74.25- 182.50) | 158.00 (87.00-301.00) |
| **LDH** | 189.00 (165.00- 263.00) | 450.00 (341.00- 597.00) | 585.50 (468.25-789.25) |
| **MG** | 1.80 (1.60- 2.00) | 2.20 (2.00- 2.40) | 2.20 (2.10- 2.40) |
| **ALB** | 4.29 (4.07- 4.64) | 4.14 (3.90- 4.40) | 3.90 (3.63- 4.20) |
| **NA** | 141.00 (139.00- 144.00) | 138.05 (136.40- 140.00) | 137.90 (135.00- 139.47) |
| **K** | 4.20 (3.80- 4.50) | 3.80 (4.10- 4.40) | 4.20 (3.80- 4.40) |

Table S2: Statistical significant correlations between studied parameters

|  | LYM | ESR | CRP | AST | ALT | LDH | G6PD | FRAP | NO | MDA | SOD | SATO2 |
| --- | --- | --- | --- | --- | --- | --- | --- | --- | --- | --- | --- | --- |
| LYM | 1 |  |  |  |  |  |  |  |  |  |  |  |
| ESR | -0.4  <0.0001 | 1 |  |  |  |  |  |  |  |  |  |  |
| CRP | -0.5  <0.0001 | +0.64  <0.0001 | 1 |  |  |  |  |  |  |  |  |  |
| AST | -0.2  <0.0001 | +0.25  <0.0001 | +0.37  <0.0001 | 1 |  |  |  |  |  |  |  |  |
| ALT | -0.2  <0.0001 | +0.18  <0.0001 | +0.30  <0.0001 | +0.86  <0.0001 | 1 |  |  |  |  |  |  |  |
| LDH | -0.4  <0.0001 | +0.37  <0.0001 | +0.47  <0.0001 | +0.62  <0.0001 | +0.57  <0.0001 | 1 |  |  |  |  |  |  |
| G6PD | +0.14  <0.0001 | -0.2  <0.0001 | -0.3  <0.0001 | -0.06  0.065 | -0.08  0.031 | -0.2  <0.0001 | 1 |  |  |  |  |  |
| FRAP | +0.22  <0.0001 | -0.3  <0.0001 | -0.4  <0.0001 | -0.2  <0.0001 | -0.2  <0.0001 | -0.2  <0.0001 | +0.14  <0.0001 | 1 |  |  |  |  |
| NO | -0.27  <0.0001 | +0.07  0.045 | +0.18  <0.0001 | +0.01  0.618 | +0.01  0.663 | +0.009  0.819 | -0.02  0.534 | -0.08  0.035 | 1 |  |  |  |
| MDA | -0.4  <0.0001 | +0.44  <0.0001 | +0.66  <0.0001 | +0.33  <0.0001 | +0.25  <0.0001 | +0.38  <0.0001 | -0.2  <0.0001 | -0.3  <0.0001 | +0.1  0.007 | 1 |  |  |
| SOD | +0.36  <0.0001 | -0.4  <0.0001 | -0.6  <0.0001 | -0.3  <0.0001 | -0.2  <0.0001 | -0.3  <0.0001 | +0.18  <0.0001 | +0.26  <0.0001 | -0.06  0.081 | -0.4  <0.0001 | 1 |  |
| SATO2 | +0.46  <<0.0001 | -0.5  <0.0001 | -0.8  <0.0001 | -0.4  <0.0001 | -0.3  <0.0001 | -0.4  <0.0001 | +0.24  <0.0001 | +0.38  <0.0001 | -0.1  <0.0001 | -0.6  <0.0001 | +0.52  <0.0001 | 1 |

Correlation coefficient, P-value
